# Weaning as Stressor for Calf Welfare

**DOI:** 10.3390/ani15091272

**Published:** 2025-04-30

**Authors:** Cecilia Guasco, Martina Moriconi, Nicoletta Vitale, Francesca Fusi, Dáša Schleicherová, Elisabetta Razzuoli, Mario Vevey, Stefania Bergagna

**Affiliations:** 1 Istituto Zooprofilattico Sperimentale del Piemonte, Liguria e Valle d’Aosta, Via Bologna 148, 10154 Torino, Italy; nicoletta.vitale@izsplv.it (N.V.); dasa.schleicherova@izsplv.it (D.S.); elisabetta.razzuoli@izsplv.it (E.R.); 2 Azienda Sanitaria Territoriale di Ancona, Viale Cristoforo Colombo 106, 60100 Ancona, Italy; martina.moriconi@sanita.marche.it; 3 Italian National Reference Centre for Animal Welfare (CReNBA), Istituto Zooprofilattico Sperimentale della Lombardia e dell’Emilia-Romagna “Bruno Ubertini” (IZSLER), Via A. Bianchi 9, 25124 Brescia, Italy; francesca.fusi@izsler.it; 4 Associazione Nazionale Bovini di Razza Valdostana, Fraz. Favret, 5, 11020 Gressan, Italy; direttore@anaborava.it

**Keywords:** weaning, stress, calves, immune response, blood, analysis

## Abstract

Weaning is a critical period for calves, affecting their well-being, health, and productivity. The present study analyzed 61 calves to evaluate the effects of weaning as a stress factor by monitoring hematological, biochemical, and immune parameters. Blood samples were collected at the start of weaning at 130–135 days (T0), 3 (T1) and 7 (T2) days later, and finally at 150 days of age. The results showed changes in the levels of cortisol, inflammatory cytokines (TNF-α, INF-γ and IL-8) and acute-phase proteins. Alterations in white blood cells were also observed, such as neutrophilia and lymphopenia, which are typical signs of an adverse stress response. Biochemically, the switch from milk to solid food caused imbalances in protein, cholesterol, and triglyceride levels, indicating a relevant metabolic adaptation. Serum electrophoresis showed a decrease in albumin and an increase in γ-globulins, a sign of immune maturation. The results suggest that weaning is one of the most important stressors for calves, with both physiological and immune effects. However, this practice is necessary for breeding productivity. Future research could investigate the role of genetics and environment in weaning resilience to further optimize breeding practices.

## Simple Summary

The weaning period is a sensitive time for calves, with implications for their well-being, health and growth. This study examined 61 Valdostana pezzata rossa and Valdostana pezzata nera-castana calves to assess the stress caused by weaning by analyzing blood, biochemical, and immune parameters. The results showed an increase in cortisol, inflammatory cytokines, and acute-phase proteins. Moreover, the results revealed alterations in white blood cells, with the typical variation in neutrophils and in lymphocytes, suggesting the adverse effects of stress. At the metabolic level, the switch from milk to solid food caused imbalances in protein, cholesterol, and triglyceride levels, indicating a metabolic adjustment. A decrease in albumin and an increase in gamma globulins, a sign of immune system maturation, were observed via serum electrophoresis. These results confirm that weaning is a significant stress factor for calves, with both physiological and immune effects, although it remains a necessary practice to ensure the reproductive efficiency of the herd.

## 1. Introduction

Animal welfare is a complex concept that concerns an animal’s ability to meet its physical and psychological needs in order to live a healthy and satisfying life. Starting with the “Five Freedoms” [1,2], the concept of animal welfare has evolved over time, developing an increasingly holistic and multidisciplinary approach. This expansion has included the concept of the “Five Provisions” [3], which emphasize the importance of providing animal subjects with adequate nutrition, a safe environment, opportunities for natural behavioral expression, positive social interactions, and optimal health conditions. An alternative means of assessing welfare was provided by the “Five Domains Model”, where wellbeing reflects a mental state domain, which is the result of four main domains of a physical and functional nature: “food”, “environment”, “health”, and “behavior” [4,5].

Acute stress can cause an acute-phase response (APR) through the release of glucocorticoids, hormones that modulate the innate immune response [6,7,8,9,10]. During the APR, pro-inflammatory cytokines and acute-phase proteins (APPs) such as haptoglobin (Hp), serum amyloid A (SA-A), C-reactive protein (CRP), and fibrinogen are produced [11]. Conversely, in situations of chronic stress, cortisol acts to suppress APR, limiting the release of pro-inflammatory cytokines, reducing antibody production by B-cells and controlling inflammation [7]. Thus, the release of glucocorticoids and catecholamines is crucial in the case of stress response, helping to inhibit pro-inflammatory mediators such as interleukin-6 (IL-6), interferon-γ (IFN-γ), and tumor necrosis factor-α (TNF-α), and regulating the physiological and behavioral changes necessary to maintain homeostasis [7,12,13]. The balance between immune suppression and activation mechanisms depends on the intensity and duration of the stress. If the release of glucocorticoids is transient, this can have adaptive effects that promote recovery, but prolonged chronic stress can lead to immune dysfunction and a weakening of the body’s defenses. Such a dual effect emphasizes the importance of monitoring and managing stress for immune health.

Weaning calves triggers behavioral and physiological responses that can negatively affect their welfare, health, and performance due to the stress experienced during this period. It has been demonstrated that the intensity of the stress response is influenced by several aspects, including age at weaning, separation patterns, and management conditions [14,15,16,17,18]. The most common behavioral manifestations include a significant increase in vocalization frequency, an increase in general activity, and in walking frequency, which indicate discomfort and difficulty in adapting to separation from the mother and to the new feeding regime [19,20,21,22]. Behavioral changes often occur simultaneously with physiological changes that can be evaluated using several biochemical and hormonal markers. For example, it has been documented that weaned calves have high levels of cortisol, the stress hormone, as well as higher levels of noradrenaline and peripheral catecholamines, which are indicators of sympathetic nervous system activation [23]. The production of APPs, such as SA-A and CRP, is also altered, indicating a systemic inflammatory response [7,24]. Other indicators, such as an altered neutrophil/lymphocyte ratio, caused by an increase in neutrophils and a decrease in lymphocytes, suggest impaired immune function during this critical period [19,23,24,25,26]. These behavioral and physiological responses highlight the complexity of the stress response in weaned calves. They underline the importance of optimizing weaning practices to minimize stress and promote better adaptation, thereby improving overall welfare, health, and long-term productivity.

The aim of the present study was to explore the impact of weaning as a stressor for the animals included in this study. This was achieved by analyzing a range of biological markers, including a complete blood count, clinical chemistry parameters, interleukins, and innate immune response throughout the weaning phase.

## 2. Materials and Methods

### 2.1. Study Design

The study was carried out on 62 calves, whose blood samples were collected at four pre-set times: before the initiation of weaning (T0) at 130–135 days of age; at 3 days after the start of weaning (T1); 7 days after the start of weaning (T2); and at the end of weaning at 150 days of age (T3). One animal died during the test.

The national association of Valdostana breeders, known as “Associazione Nazionale Allevatori Bovini di Razza Valdostana” (A.N.A.Bo.Ra.Va.) in Italian, is head-quartered in Gressan, in the province of Aosta. The association is involved in many activities, including the genetic enhancement of the Valle d’Aosta cattle breed, the evaluation of reproductive specimens, the organization of national and international exhibitions, the administration of the Genetic Center for performance assessments, and the supervision of the bull center, where the semen of bulls utilized for artificial insemination is produced.

Calves are admitted to the Genetic Centre at the age of 45–60 days. In accordance with the prevailing health regulations, they are held in a quarantine stall to prevent contact with the other calves at the Genetic Centre. During this initial period, which presents certain challenges due to the difficulties of adapting to the new surroundings, group cohesion, and nutritional intake, the subjects are provided with particular care and support to facilitate the process of weaning, which typically occurs at approximately 4 months of age. Furthermore, the incidence of the most prevalent infectious diseases, such as bovine tuberculosis (TB) and bovine infectious rhinotracheitis (IBR), are also examined. Calves were housed in ten pens, each with an indoor area (5 × 5 meters) and an outdoor area (4 × 4 meters). Each pen housed eight animals and was equipped with two drinking devices. The bedding was permanent: it was completely replaced every 21 days, while chopped straw was added daily to ensure cleanliness and comfort. In order to maintain homogeneous environmental conditions, all calves received the exact same diet according to the entry cycle. The quantity of milk distributed to each calf decreased progressively with age, from 8.00 kg/day at the age of 45–60 days to 0.00 kg/day at 130–135 days, while the quantity of feed distributed to each calf increased progressively with age, from approximately 2.5 kg per day at the time of entry into the Genetic Centre to approximately 4.5 kg per day at the conclusion of the test at 11 months of age. The distribution of feed was regulated by an automated feeder, which utilizes a magnetic collar to identify each calf. In addition to the feed, the calves were provided with hay ad libitum.

### 2.2. Blood Analysis

Hematological analyses were performed on whole-blood samples collected in Vacutainer tubes containing the anticoagulant K_3_EDTA (purple cap), while serological analyses were performed on serum samples obtained using tubes without an anticoagulant (red cap). The tubes for serological analyses were centrifuged at 3500 rpm for 15 min at 20 °C and the serum was stored in 1.5 mL aliquots at −80 °C until analysis.

The investigations included the performance of complete blood counts, clinical chemistry, protein electrophoresis, innate immunity (lysozyme, complement titration, bactericidal, IFN-γ and TNF-α) and pro-inflammatory cytokines (interleukin-8). Of the 62 calves identified at the beginning of the study, diagnostic tests were carried out on 61 animals at the four scheduled times, resulting in a total of 2196 laboratory tests.

#### 2.2.1. Complete Blood Count (CBC) Analysis

Blood samples containing anticoagulant K_3_EDTA were immediately analyzed for hematocrit measurements using the appropriate instrumentation (MS4 instrument, Wefen company, Milan, Italy), in accordance with the manufacturer’s instructions.

The parameters evaluated in the CBC analysis were as follows: erythrocytes (RBC), hemoglobin (HB), hematocrit (HCT), mean cell volume (MCV), mean corpuscular hemoglobin (MCH), mean corpuscular hemoglobin concentration (MCHC), leucocytes (WBC) with leucocyte formula (lymphocytes (LINF), monocytes (MON), neutrophils (NEU), eosinophils (EO) and basophils (BA)) and platelets (PLT). The reference values employed for the analytical procedures were those established using the automated analyzer with the appropriate methodology before validation.

#### 2.2.2. Biochemical Analysis

Prior to the analysis, serum samples previously stored at −80 °C were set up at room temperature and then mixed for a few seconds on a vortex mixer, taking care to avoid foam formation. This step was carried out with the utmost care before proceeding with testing. The serum aliquots were analyzed using an automated photometer (BT 1500 vet, Futurlab-Instrumentation Laboratory, Padua, Italy) in compliance with the manufacturer’s instructions. A comprehensive range of biochemical parameters was examined, including alkaline phosphatase (ALP), alanine aminotransferase (ALT), aspartate aminotransferase (AST), calcium (Ca), cholesterol (CHOL), creatinine (Crea), iron (Fe), gamma-glutamyl transferase (GGT), chlorine (Cl), magnesium (Mg), phosphorus (P), total protein (TP), triglycerides (TG), and urea (UREA). The reference values employed for the analyses were obtained from the bibliography [27].

#### 2.2.3. Serum Protein Electrophoresis

The assessment of serum proteins was performed using previously stored serum samples. The analysis involved a zonal electrophoresis of the serum proteins and was performed by separating the fractions based on their net electrical charge in an alkaline medium (pH 9.2) on a 0.8% agarose gel, which was then stained with an Amidoscharwz solution. The test was carried out using a semi-automatic multi-parametric biochemical analyzer for electrophoresis (HYDRASYS LC SEBIA, HYGRAGEL PROTEIN 15/30 kit and densitometer). After electrophoresis, the serum proteins were separated into four distinct fractions, as illustrated in the proteinogram: albumin (ALB), α-globulins (α-G), β-globulins (β-G), and γ-globulins (γ-G).

#### 2.2.4. Immunoenzymatic Analysis

Quantitative analyses of the plasma levels of IFN-γ, TNFα, and IL-8 were conducted using commercially available kits, following the standard protocols and instructions provided by the manufacturer. In particular, the following enzyme-linked immunosorbent assay (ELISA) kits were used:

- “Bovine Interferon-γ ELISA Kit” (Standard curve range: 1–1000 pg/mL; Sensitivity: 0.5 pg/mL), MABTECH AB, (Nacka Strand, Sweden);
- “Bovine Tumor Necrosis Factor Alpha ELISA Kit” (Standard curve range: 10–3000 ng/L; Sensitivity: 5.56 ng/L), Bioassay Technology Laboratory (Shanghai, China);
- “Bovine Interleukin 8 ELISA Kit” (Standard curve range: 5–1000 ng/L; Sensitivity: 2.39 ng/L), Bioassay Technology Laboratory (Shanghai, China).

#### 2.2.5. Lysozyme Titration

A lysozyme titration was conducted on previously prepared and frozen blood serum. The serum sample was placed in contact with a *Micrococcus lysodeikticus* (ATCC 4698), a microorganism that is particularly sensitive to the lytic activity of lysozyme, incorporated in an agar gel. The presence of lysozyme was confirmed following an 18 h incubation at 37 °C in a humid chamber, during which a halo of germ lysis was observed around the sample deposition well. The concentration of lysozyme was determined by measuring the diameter of the clarification ring observed around the well with a caliper, with the result proportional to the concentration of lysozyme present. This was achieved by incubating known amounts of lysozyme and establishing a standard curve [28].

#### 2.2.6. Serum Bactericide

The frozen serum sample was cultured in 96-well micromethod plates with a known amount of *E. coli* in a suitable nutrient medium (simple broth) and support medium (Veronal buffer). The bactericidal capacity of the serum was finally measured by comparing the variation between the turbidity of the culture wells in the presence and absence of the test serum, measured by reading the optical density with a spectrophotometer (690 nm). Results are expressed as the percentage (%) of bactericidal activity of the test serum [29].

#### 2.2.7. Complement Titration

The test is based on the quantification of the lytic activity of the serum against rabbit red blood cells (activation of the alternative complement pathway).

In brief, 5 mL of rabbit blood is taken with a sterile syringe, diluted in 5 mL of Alsever solution, and centrifuged at 2000 rpm for 10 min. At the end of centrifugation, the supernatant is removed and the precipitate washed with Veronal buffer three times. Finally, a 3% rabbit red blood cell suspension is prepared and incubated at 37 °C ± 2 °C for 30 min before continuing with the next steps of the analysis. In a 96-well plate, 150 µL of distilled water is placed in row 1, 150 µL of Veronal buffer and 50 µL of test serum are placed in row 2, and150 µL of Veronal buffer is placed in row 12. Then, 100 µL of Veronal buffer is dispensed into rows 3, 4, 5, 6, 8, 9, 10, and 11. Subsequently, 100 µL of a solution consisting of Veronal buffer and test serum is taken from row 2 and transferred to row 3, then from row 3 to row 4, and so on, until row 6, discarding the last 100 µL taken. The same procedure is also performed for rows 8, 9, 10, and 11. At this point, 50 µL of Veronal buffer and 25 µL of rabbit red blood cell are added to the same rows as previously treated (3, 4, 5, 6, 8, 9, 10, and 11). Finally, the plate is shaken gently and incubated at 37 °C ± 2 °C for 30 min. Then, 100 µL of the supernatant from each 96-well plate is transferred to a new plate to measure the optical density using a spectrophotometer at a wavelength of 550 nm [29].

### 2.3. Statistical Analysis

The sample size was calculated for an analysis of variance (ANOVA) study design with repeated measures within factors using G*Power (Version 3.1.9.7) with the following settings: an alpha error of 0.05, an effect size of 0.25, a power of 0.95, a correlation among repeated measures of 0.5 and four time points (T0, T1, T2, and T3). All data for blood count, clinical chemistry, interleukins, and the innate immune response are presented as the mean ± standard deviation for descriptive purposes. A statistical standardization process (mean = 0, standard deviation = 1) was applied to the immunoenzymatic parameters to facilitate their visual comparison on a single graph across the four time points (T0, T1, T2, T3).

Since the Shapiro–Wilk test revealed that the dependent variables were not distributed normally, the comparison between the four moments, for each parameter, was performed via a non-parametric analysis of variance using the Kruskal–Wallis chi-square test on the median. When the data collected did not follow a normal distribution, the comparison between the four time points (T0, T1, T2, and T3) was carried out using a non-parametric analysis of variance, in particular the chi-square test on the median. This test allows us to check whether the medians of the values observed at different times are significantly different from each other. In other words, we assessed whether the variations observed in the blood parameters during the weaning period were attributable to chance or represented a true biological change.

## 3. Results

### 3.1. CBC Analysis

The blood count showed variations in some parameters at different sampling times.

The mean values obtained from the CBC analysis are reported in Table 1.

The mean values of lymphocytes and neutrophils had a non-normal distribution, so we used a test with a non-parametric analysis of variance: specifically, the chi-square test on the median. The lymphocyte values at T1 and T2 showed lower median values (T1: 48.2, T2: 47.1) than before (T0: 52.5) and after weaning (T3: 51.0). This difference was found to be statistically significant (chi-squared 9.5, *p* = 0.024). Neutrophil values at three (T1: 43.2) and seven (T2: 45.2) days following the commencement of weaning exhibited higher median values in comparison to those recorded before weaning (T0: 39.8) and after weaning (T3: 37.9), with a statistically significant difference (chi-squared 8.6, *p* = 0.034).

The monocyte, mean platelet volume, and red blood cell distribution width values were always slightly above the reference ranges at the four points analyzed. In contrast, the values for eosinophils and mean cell volume were always lower than the reference ranges.

### 3.2. Biochemical Analysis

Observations of the biochemical parameters analyzed revealed some relevant alternations of values in the animals’ metabolism. In particular, the aspartate transaminase values were always below the reference ranges at all time points, including T0, as were the calcium and the chlorine value. Phosphorus and gamma-glutamyl transferase values were always above the reference ranges during the sampling points. The cholesterol level decreased further at T2 and then increased again at T3. Total protein values remained below the reference ranges, then increased in the last sampling period (T3), as creatinine did. Triglyceride values were within the range for the first two samples, but then these values increased again at T2 and T3. The results of the mean values for the biochemical analysis are shown in Table 2.

### 3.3. Serum Protein Electrophoresis

Not all parameters showed values within the ranges at all sampling times. In particular, albumin and gamma globulin were below the ranges, whereas alpha- and beta-globulins were above the ranges. Mean of electrophoresis parameters over the 4 study times are shown in Table 3.

### 3.4. Immunoenzymatic Analysis

BA, LIZ, and TC values were always in the defined ranges. The value of TNF-α decreased slightly at T1 but increased significantly at T2 and especially at T3. As for IL-8, the value decreased at T1 and continued to decrease at T2, but, at T3, this parameter approached the level of the reference value. All parameters showed a non-normal distribution. The mean values of the interleukins analyzed at the four study time points are shown in Table 4.

Concerning the bactericidal values, it was observed that, as the number of days during the weaning phase increased, the values rose from a median value of 91.3 (time T0) to a value of 92.5 at time T3. This difference was statistically significant (*p*-value < 0.05). The median IFN-γ values showed a fluctuating trend over time: they decreased at T1 (median 9.4), subsequently increased at T2 (13.2), and then decreased again at T3 (12.2). The statistical analysis showed a significant difference between the different time points (*p*-value < 0.05). Otherwise, a decrease in median IL-8 values was observed from time T0 (median 148.9) to time T2 (median 121.6); then, an increase in this parameter was observed at T3 (137.8). The difference during the observed times was statistically significant (*p*-value < 0.05). The TNF-α values changed in terms of median values over time. In particular, from T1 (median 145.7) to T3 (median 344.1), a statically significant (*p*-value < 0.05) increase was observed. Finally, compared to the other two parameters of complement titration and lysozyme, no change in values was observed over time and the values remained constant.

## 4. Discussion

Weaning is not simply the removal of milk from the calves’ diet and their physical separation from their mothers; it is a complex process involving many significant changes in the animals’ lives. During this period, calves are exposed to a variety of new experiences that can be destabilizing, e.g., the reorganization of social relationships within the group as they are introduced to new social contexts, and changes in housing arrangements, which may involve adapting to different spaces or new types of housing. These changes are potential sources of stress for calves, who face simultaneous physical, social, nutritional, and psychological stimuli. The stress arising from these situations can have a negative impact on the animals’ overall health, particularly on their immune systems. A compromised immune system makes calves more susceptible to neonatal diseases and infections, which can affect their growth, overall welfare, and, ultimately, production performance.

Although weaning is considered a major source of stress for beef calves, it is a practice that is essential to ensuring their reproductive efficiency. In fact, the early separation of calves accelerates the resumption of the reproductive activity of their mothers, favoring a shorter interval between calvings and thus improving the overall productivity of the herd. The hemochromocytological analyses carried out on the animals did not reveal any deviation of the parameters from the reference ranges, with the exception of monocytes, since significant changes were observed in two parameters in particular, neutrophils and lymphocytes: the characteristic stress leukogram, represented by neutrophilia, and lymphopenia, observed from T0 to T2, i.e., in the very first days after weaning. It is known that, mainly due to changes in subpopulations of these cells, particularly neutrophils, the total number of leukocytes increases during weaning and introduction into a new housing situation [22,25]. Concomitantly, stressful events are associated with a decrease in lymphocyte subsets, a phenomenon that could be related to a redistribution of these cells from peripheral blood vessels to immune compartments and tissues [22].

Almost all the chemical–clinical parameters analyzed showed significant alterations during the weaning period, revealing the profound impact that this phase has on calves’ lives. These changes are mainly attributable to the radical change in the diet to which the animals are subjected, from a diet exclusively based on milk, which is easily digestible and highly nutritious, to a diet composed entirely of solid foodstuffs, which required the physiological and metabolic adaptation of the digestive system. This transition involves not only a change in the composition of ingested nutrients but also the activation and maturation of specific digestive and fermentative mechanisms necessary for the management of a solid diet. Consequently, significant changes in biochemical and clinical parameters are observed, which are indicators of metabolic stress and adaptation to this new growth phase. Such alterations are an evident indication of the relevant changes that calves go through during weaning, not only from a nutritional point of view, but also physiologically and immunologically. Furthermore, our results are also supported by previous studies that have shown that dietary problems can cause electrolyte imbalances in the blood [30]. A previous study showed similar changes in some parameters in the post-weaning period. In particular, values below the reference ranges were found for AST, creatinine (Crea), cholesterol (Chol) and total protein (TP). Although in adult cattle such variations may indicate disease or the dysfunction of organs such as the liver or kidney, in clinically healthy calves, values outside the reference ranges may be related to feeding [31]. Cholesterol levels increase during the milk feeding period and then decrease after weaning [32,33]. Increased triglyceride levels may indicate that the calves received a high-energy post-weaning diet [31]. Out-of-range levels of calcium and phosphorus may be related to the type of feed given to calves [34]. Furthermore, higher phosphorus levels are associated with higher concentrations of growth hormone, which stimulates renal reabsorption of phosphate [27]. Changes in muscle mass, which are typical of growth, may be associated with reduced serum creatinine concentrations [34].

The electrophoretic profile revealed marked alterations in the concentrations of all the protein fractions analyzed, except for beta globulins, which remained unchanged. These significant alterations in the electrophoretic pattern highlight the profound physiological adjustments that occurred during the observation period. Albumin is the main protein responsible for the maintenance of oncotic pressure in plasma, and altered values may indicate a compromised physiological state [35,36]. However, the increased serum of albumin, total protein, and globulin concentrations could be attributed to an increased intake of total crude protein due to increased solid feed consumption, as previously demonstrated [37]. Regarding α-globulins, environmental conditions are an important factor that can influence acute phase protein levels. It is noteworthy to mention that elevated α-globulins are not necessarily associated with inflammatory disease and may occur in the absence of disease. Therefore, higher relative concentrations of α-globulins in calves should not be automatically interpreted as a sign of the activation of inflammatory processes or a pathological condition [38]. The stability of β-globulins indicates the lower susceptibility of this fraction to the factors influencing the other protein components. Finally, the progressive increase in γ-globulin concentrations in calves with increasing age has been shown to be closely related to the normal growth process and maturation of the immune system [38].

As assessing welfare remains a key challenge in calf production, special emphasis was placed on stress markers. Regarding interleukins and innate immunity, the present study confirmed the results already reported in the literature and showed an increase in the levels of some key cytokines at the end of the withdrawal times [25,26]. Significant increases were observed in TNF-α, INF-γ and IL-8, key molecules in the regulation of immune responses. TNF-α is known to play a central role in mediating inflammation and response to pathogens [39], while INF-γ is critical for macrophage activation and the modulation of adaptive immune responses [40]. IFN-γ mediates its protective effects by triggering the activation of lysosomal activity, inducing nitric oxide production and the expression of effector genes such as immunity-related GTPases, as well as modulating the metabolic activity of antigen-presenting cells, including dendritic cells and macrophages [41]. IL-8 is a pro-inflammatory cytokine that promotes the recruitment of neutrophils to sites of infection or tissue damage. The increase in these cytokines observed in calves may reflect an activation of innate immunity in response to environmental, nutritional, or management stimuli during the weaning period, a critical time for immune system development and maturation.

Further evidence of the activation of a physiological response to a stressful event, such as weaning, is provided by the observed changes in bactericidal, lysozyme, and complement levels. Lysozyme is an antimicrobial protein present in many body fluids. It plays an essential role in the first line of defense against bacterial infection by degrading the bacterial cell wall [42]. Its increased levels suggest increased innate immune activity to counteract pathogens at this critical stage. Similarly, complement is a key component of the innate immune system. It is responsible for the direct destruction of pathogens and promotes phagocytosis [43]. The increase in its levels reflects an adaptive response by the organism to the challenges of weaning, which include social, nutritional, and environmental stresses. Finally, bactericides, an indicator of the body’s ability to neutralize pathogenic micro-organisms, show a significant increase, confirming the activation of defense mechanisms. This complex set of immune responses demonstrates how the weaning period intensely stimulates the immune system and highlights the impact that stressful events can have on the physiology and well-being of animals.

## 5. Conclusions

The study highlights the physiological and immunological changes that occur in calves during weaning, a critical period in their development. The data confirm that weaning is a complex and multifaceted stressor that induces adjustments in both metabolic and immune functions. The observed alterations in hematological and biochemical parameters underline the activation of stress responses.

Although weaning is undoubtedly a significant source of stress for calves, it remains a necessary practice to ensure the reproductive efficiency of the herd and improve overall productivity.

Future research should explore the genetic and environmental factors that influence individual resilience to weaning stress, paving the way for more tailored management practices. By integrating this knowledge, it will be possible to further optimize animal welfare while maintaining the productivity of livestock farms.

## Figures and Tables

**Table 1:** Means ± SD of the CBC parameters at the four time points.

Parameter	Reference Values	T0	T1	T2	T3
BA	/	0.416 ± 0.033	0.509 ± 0.033	0.409 ± 0.032	0.455 ± 0.036
EO (%)	2–20%	0.360 ± 0.143	0.357 ± 0.089	0.273 ± 0.046	0.542 ± 0.150
WBCs	4–12 m/mm^3^	11.96 ± 0.70	10.63 ± 0.38	11.62 ± 0.46	11.34 ± 0.46
RBCs	6–11 m/mm^3^	9.10 ± 0.14	9.22 ± 0.12	9.42 ± 0.12	9.22 ± 0.14
HB	8–15 g/dL	11.50 ± 0.19	11.60 ± 0.16	11.89 ± 0.15	11.55 ± 0.17
HCT	25–50%	28.89 ± 0.51	29.45 ± 0.40	30.13 ± 0.44	29.54 ± 0.50
LINF	45–75%	52.60 ± 1.21	47.58 ± 1.21	48.26 ± 1.15	50.96 ± 1.07
MCH	11–17 pg	12.60 ± 0.108	12.54 ± 0.103	12.60 ± 0.097	12.50 ± 0.097
MCHC	30–40 g/dL	39.89 ± 0.28	39.35 ± 0.24	39.54 ± 0.28	39.43 ± 0.27
MCV	40–60 fl	31.80 ± 0.30	32.05± 0.29	32.06 ± 0.29	31.95 ± 0.27
MON	1–5%	5.84 ± 0.15	6.61 ± 0.16	6.26 ± 0.17	5.47 ± 0.15
MPV	3–8 fl	9.87 ± 0.10	9.82 ± 0.09	9.90 ± 0.09	9.77 ± 0.09
NEU	15–47%	40.77 ± 1.18	44.93 ± 1.21	44.77 ± 1.13	42.58 ± 1.09
PCT	/	0.537 ± 0.04	0.538 ± 0.037	0.554 ± 0.032	0.508 ± 0.030
PDW	6–10 fl	9.05 ± 0.24	9.26 ± 0.21	9.21 ± 0.20	9.41 ± 0.16
PLT	100–800 m/mm^3^	543.98 ± 36.67	556.55 ± 35.35	552.68 ± 29.03	528.40 ± 29.16
RDW	8–12%	17.20 ± 0.25	17.19 ± 0.26	16.98 ± 0.24	16.93 ± 0.19
RRg	/	1.07 ± 0.50	1.16 ± 0.51	0.91 ± 0.38	1.42 ± 0.64

“BA”: basophil; “EO”: eosinophil; “WBCs”: leukocytes; “RBCs”: erythrocytes; “HB”: hemoglobin; “HCT”: hematocrit; “LINF”: lymphocytes; “MCH”: mean corpuscular hemoglobin; “MCHC”: mean corpuscular hemoglobin concentration; “MCV”: mean cell volume; “MON”: monocytes; “MPV”: mean platelet volume; “NEU”: neutrophils; “PCT”: platelet crit; “PDW”: platelet distribution width; “PLT”: platelets; “RDW”: red blood cell distribution width; “RRg”: reticulocytes. “T0”: before the initiation of weaning at 130–135 days of age; “T1”: at 3 days after the start of weaning; “T2”: 7 days after the start of weaning; “T3”: at the end of weaning at 150 days of age. “/”: reference range of values not available.

**Table 2:** Means ± SD of the biochemical parameters at the four study points.

Parameter	Reference Values	T0	T1	T2	T3
ALP	0–488 U/L	204.65 ± 10.33	* 192.49 ± 9.4	* 155.81 ± 6.49	* 167.88 ± 7.33
ALT	11–40 U/L	15.01 ± 0.50	*** 18.44 ± 0.70	*** 16.80 ± 0.63	*** 21.09 ± 0.65
AST	78–132 U/L	64.36 ± 1.99	** 73.19 ± 2.43	** 61.60 ± 1.66	** 72.14 ± 1.72
CA	9.7–12.4 mg/dL	8.62 ± 0.055	*** 8.74 ± 0.11	*** 7.84 ± 0.10	*** 8.81 ± 0.057
CHOL	80–120 mg/dL	79.78 ± 2.08	* 81.88 ± 2.21	* 73.08 ± 2.32	* 80.44 ± 1.63
CL	97–111 mmol/L	91.42 ± 0.50	** 95.49 ± 0.72	** 92.08 ± 0.50	** 93.54 ± 0.59
CREA	1–2 mg/dL	0.88 ± 0.023	*** 0.95 ± 0.023	*** 0.83 ± 0.022	*** 1.14 ± 0.029
FE	57–162 ug/dL	110. 85 ± 5.81	*** 88.80 ± 5.94	*** 111.80 ± 4.17	*** 127.47 ± 5.53
GGT	6.1–17.4 U/L	19.75 ± 0.78	19.86 ± 0.71	18.21 ± 0.76	19.54 ± 0.90
MG	1.8–2.3 mg/dL	2.09 ± 0.026	*** 2.38 ± 0.039	*** 2.03 ± 0.034	*** 2.27 ± 0.026
PHOS	5.6–6.5 mg/dL	9.32 ± 0.15	* 8.86 ± 0.16	* 8.83 ± 0.13	* 9.06 ± 0.13
TP	6.74–7.46 g/dL	6.27 ± 0.077	*** 6.48 ± 0.068	*** 5.98 ± 0.089	*** 6.85 ± 0.070
TRIG	0–14 mg/dL	14.09 ± 0.95	** 14.29 ± 0.92	** 17.40 ± 0.83	** 18.27 ± 1.01
UREA	20–30 mg/dL	21.04 ± 0.63	* 20.39 ± 0.58	* 23.04 ± 1.10	* 23.49 ± 0.77

“ALP”: alkaline phosphatase; “ALT”: alanine aminotransferase; “AST”: aspartate transaminase; “CA”: calcium; “CHOL”: cholesterol; “CL”: chlorine; “CREA”, creatinine; “FE”: iron; “GGT”: gamma-glutamyl transferase; “MG”: magnesium; “PHOS”: phosphorus; “TP”: total protein; “TRIG”: triglycerides; “UREA”: urea. * *p* < 0.05, significant difference with respect to T0. ** *p* < 0.005, significant difference with respect to T0. *** *p* < 0.0001, significant difference respect to T0. “T0”: before the initiation of weaning at 130–135 days of age; “T1”: at 3 days after the start of weaning; “T2”: 7 days after the start of weaning; “T3”: at the end of weaning at 150 days of age.

**Table 3:** Means ± SD of electrophoresis parameters at the four time points.

Parameter	Reference Values	T0	T1	T2	T3
ALB	3.03–3.55 g/dL	2.63 ± 0.037	* 2.89 ± 0.025	* 2.46 ± 0.058	* 3.01 ± 0.037
α-G	0.75–0.88 g/dL	1.29 ± 0.021	* 1.26 ± 0.019	* 1.22 ± 0.022	* 1.43 ± 0.034
β-G	0.8–1.12 g/dL	1.21 ± 0.025	1.22 ± 0.034	1.14 ± 0.023	1.09 ± 0.042
γ-G	1.69–2.25 g/dL	1.13 ± 0.048	* 1.10 ± 0.033	* 1.15 ± 0.036	* 1.29 ± 0.041

“ALB”: albumin; “α-G”: alpha globulin; “β-G”: beta globulin; “γ-G”: gamma globulin. * *p* < 0.05, significant difference with respect to T0. “T0”: before the initiation of weaning at 130–135 days of age; “T1”: at 3 days after the start of weaning; “T2”: 7 days after the start of weaning; “T3”: at the end of weaning at 150 days of age.

**Table 4:** Means ± SD of immunoenzymatic analysis parameters at the four study time points.

Parameter	Reference Values	T0	T1	T2	T3
INF-γ		13.85 ± 1.59	11.61 ± 0.87	15.09 ± 0.96	15.17 ± 1.33
BA	>90%	91.04 ± 0.29	91.53 ± 0.22	91.85 ± 0.29	92.08 ± 0.37
LIZ	1–3 μg/mL	1.47 ± 0.055	1.63 ± 0.064	1.63 ± 0.060	1.66 ± 0.068
TC	>30 CH50/150 µL	47.31 ± 1.52	48.54 ± 1.55	48.41 ± 1.65	47.41 ± 1.44
TNF-α		229.80 ± 15.66	220.40 ± 20.16	299.92 ± 20.53	335.27 ± 22.80
IL-8		148.46 ± 5.38	141.79 ± 10.22	124.78 ± 7.13	148.32 ± 10.30

“INF-γ”: interferon-gamma; “BA”: bactericide; “LIZ”: lysozyme; “TC”: completion title; “TNF-α”: tumor necrosis factor; “IL-8”: interleukin 8. “T0”: before the initiation of weaning at 130–135 days of age; “T1”: at 3 days after the start of weaning; “T2”: 7 days after the start of weaning; “T3”: at the end of weaning at 150 days of age.

## Data Availability

The data presented in this study are available on request from the corresponding author.
